# Formal Public Health Education and Career Outcomes of Medical School Graduates

**DOI:** 10.1371/journal.pone.0039020

**Published:** 2012-06-20

**Authors:** Marie Krousel-Wood, Jiang He, Meredith Booth, Chung-Shiuan Chen, Janet Rice, Marc J. Kahn, Rika Maeshiro, Paul K. Whelton

**Affiliations:** 1 Department of Epidemiology, Tulane University School of Public Health and Tropical Medicine, New Orleans, Louisiana, United States of America; 2 Department of Family and Community Medicine, Tulane University School of Medicine, New Orleans, Louisiana, United States of America; 3 Center for Health Research Ochsner Clinic Foundation, New Orleans., Louisiana, United States of America; 4 Comprehensive Alcohol Research Center, Louisiana State University Health Sciences Center, New Orleans, Louisiana, United States of America; 5 Department of Biostatistics, Tulane University School of Public Health and Tropical Medicine, New Orleans, Louisiana, United States of America; 6 Department of Medicine, Tulane University School of Medicine, New Orleans, Louisiana, United States of America; 7 Public Health and Prevention Projects, Association of Academic Medical Colleges, Washington D.C, United States of America; Medical Research Council South Africa, South Africa

## Abstract

**Background:**

Few data are available evaluating the associations of formal public health education with long-term career choice and professional outcomes among medical school graduates. The objective of this study was to determine if formal public health education via completion of a masters of public health (MPH) degree among US medical school graduates was associated with early and long-term career choice, professional satisfaction, or research productivity.

**Methods:**

We conducted a retrospective cohort study in 1108 physicians (17.1% completed a MPH degree) who had 10–20 years of follow-up post medical school graduation. Multivariable logistic regression analyses were conducted.

**Results:**

Compared to their counterparts with no MPH, medical school graduates with a MPH were more likely to have completed a generalist primary care residency only [relative risk (RR) 1.79, 95% confidence interval (CI) 1.35–2.29], obtain employment in an academic institution (RR 1.81; 95% CI 1.33–2.37) or government agency (RR 3.26; 95% CI 1.89–5.38), and practice public health (RR 39.84; 95% CI 12.13–107.38) or primary care (RR 1.59; 95% CI 1.18–2.05). Furthermore, medical school graduates with a MPH were more likely to conduct public health research (RR 8.79; 95% CI: 5.20–13.82), receive NIH or other federal funding (RR 3.11, 95% CI 1.74–5.33), have four or more peer-reviewed publications (RR 2.07; 95% CI 1.56–2.60), and have five or more scientific presentations (RR 2.31, 95% CI 1.70–2.98).

**Conclusion:**

Formal public health education via a MPH was associated with career choice and professional outcomes among physicians.

## Introduction

It is well-recognized that public health and medicine must work together in an integrated model if we are to train the best possible healthcare workforce, develop innovative tools and approaches through research, and ultimately achieve the maximum potential for improving health [1]. A series of reports in the United States (US) and internationally highlight the importance of public health education and training for physicians in preparing them to address the complex challenges of the 21^st^ century [2]–[7]. Several countries have identified shortfalls in public health training and continued opportunities for creating important linkages between medical education and public health [8]–[13]. Common approaches to providing formal public health training for physicians in the US include undergraduate training (via combined Medical Doctor/Masters of Public Health-MD/MPH programs), residency programs (such as General Preventive Medicine) and mid-career completion of a MPH. The Institute of Medicine has recommended that a significant proportion of medical school graduates be fully trained in the ecologic approach to public health at the MPH level [3] and that both formal training (at the master’s level) for physicians pursuing public health careers and continuing public health education for all practicing physicians regardless of their specialty be provided [4].

Despite the growing interest in and need for public health education, there is limited evidence that formal public health education via completion of a MPH degree among US medical school graduates is associated with early and long-term career choice, professional satisfaction, or research productivity. Prior research has been limited by small sample sizes, short periods of follow-up, and lack of data from a comparison group [14]–[16]. We hypothesized that career choice and professional development and long-term outcomes (e.g, professional position, type of medical practice, and scientific publications) would differ among medical school graduates with versus without a MPH. To address this hypothesis, we conducted a retrospective cohort study of physicians including a relatively large group completing formal public health education (i.e., MPH degree) [17] and a comparison group without a MPH degree 10–20 years after graduation from a US medical school.

## Methods

The study was approved by Tulane University’s Institutional Review Board. A waiver of written informed consent was granted for this minimal risk survey study.

We studied a cohort of physicians (n = 1783) who graduated from Tulane University School of Medicine between 1985 and 1997 and were identified by means of the American Medical Association Physician Professional Data provided by Axciom Corporation and J. Knipper and Company, Inc. The physicians who completed a MPH (defined as a Masters of Public Health (MPH), Masters of Science and Public Health (MSPH), or Masters of Public Health and Tropical Medicine (MPH&TM) were identified using the administrative databases maintained at the school and responses to the study survey. A majority of these physicians earned a general public health degree at the Tulane School of Public Health and Tropical Medicine, completing 36 credit hours with at least 15 credits of required core public health courses including biostatistics, epidemiology, health systems management, environmental health sciences, and social and behavioral influences on health.

The survey was conducted between October 2007 and May 2009. A survey packet was mailed to each graduate and included a cover letter with a request for a current résumé and details regarding the modest incentive ($20 gift card) for participating, the survey, and a postage paid envelope [18]. For those graduates who did not respond or whose survey was undeliverable, addresses were verified using Google search, online White Pages search and physician searches using www.vitals.com, www.healthgrades.com, and www.finddoctors.org. Up to 12 mailings were sent to each graduate. The survey included questions regarding demographics, residency training, professional satisfaction and career activities including research.

Demographic data were collected from administrative records in the school of medicine: age at the time of medical school graduation, gender, and self-reported race (white, black, Hispanic/Latino, Asian or Pacific Islander, American Indian or Alaska Native or other). Information on race was collected in an effort to describe the demographic diversity of the graduates. Based on distribution of the data, race was categorized as white versus non-white, undergraduate university region as southern versus other (including northeast, mid west, west coast and outside US), undergraduate major as science versus non-science. Overall undergraduate grade point average (on a 4.0 scale) was categorized as <3.4 and ≥3.4 based on the mean grade point average and Medical College Admission Test (MCAT) scores as <30 and ≥30 based on a cut point associated with academic performance [19]. Time since graduation from medical school (in years) was calculated using the date of medical school graduation and the date of survey completion (categorized as <15 years and ≥15 years).

Early career activities included induction into the Alpha Omega Alpha medical honor society which was obtained from the records administrator at Alpha Omega Alpha’s national office in Menlo Park, CA. Graduate medical education including residency and fellowship training information was collected from the survey and participants were grouped into one of two categories: a) generalist primary care only: general internal medicine, general pediatrics, and family medicine or b) specialty care: all surgical specialties including obstetrics and gynecology, all medical and pediatric subspecialties, anesthesiology, radiology, psychiatry, ophthalmology, dermatology, emergency medicine, pathology, neurology, preventive medicine, and other. Board certification status was obtained through the American Board of Medical Specialties online profile service (https://profileservice.abms.org).

Professional practice and achievements 10–20 years after medical school graduation were collected from the survey, which allowed for multiple response options given the varied professional settings of the respondents. Employment variables included *type of organization* and *professional position*. Medical practice variables included *type of medical practice*; *practice setting*; *US practice region*; and *medical care of underserved patients*. Time spent on various professional activities included *time spent on patient care, administrative activities, research and teaching*. Professional satisfaction variables included *satisfaction with career path* and *net annual taxable income*. Research activity variables included *type of research*, *research funding sources*, and *receipt of research funding* as a lead investigator. The number of scientific presentations and peer-reviewed and other publications reported were categorized based on distribution of the data in the entire sample.

### Statistical Analysis

All data were double entered and discrepancies were corrected using primary source data. Baseline characteristics, early career activities, and long-term professional achievements including research were compared between physicians who did and did not complete a MPH using Χ^2^ and t tests where appropriate. Graduates who did not complete a MPH included those who completed the MD only and those who completed a MD/degree combination other than the MPH.

To determine the association between MPH education and early career activities, multivariable logistic regression analyses (adjusted for age, race, gender, and differences in baseline characteristics including undergraduate university region and time since graduation) were conducted for each outcome. In the multivariable models, we examined interactions between time since graduation and each outcome to determine if the results should be stratified by time since graduation (both as a continuous variable and cut point of < or ≥15 years). Because the interaction between each study outcome and time since graduation had p values >0.05, we did not stratify the multivariate models.

Because the rate of early career achievement outcome was common (>10% for each outcome), we approximated relative risk (RR) from the adjusted odds ratio (using a published method of correcting the odds ratio in cohort studies of common outcomes [20]) to derive an estimate of the association between MPH education on each outcome.

In assessing the relationship between MPH education and professional achievements 10–20 years after medical school graduation, we used multinomial logistic regression when the outcome variables had multiple levels. Adjustments were made for differences in age, race, gender, undergraduate university region, time since graduation, generalist primary care training, employment by an academic institution and faculty or research appointment. [Adjustments for employment in academic institutions and faculty appointments were done for the analyses of medical practice outcomes and research activities]. Risk ratios were approximated using the approach previously described. Less than 10% of the data were missing for each outcome variable. All analyses were performed using SAS version 9.2 (SAS Institute, Cary, North Carolina).

## Results

**Figure 1:**
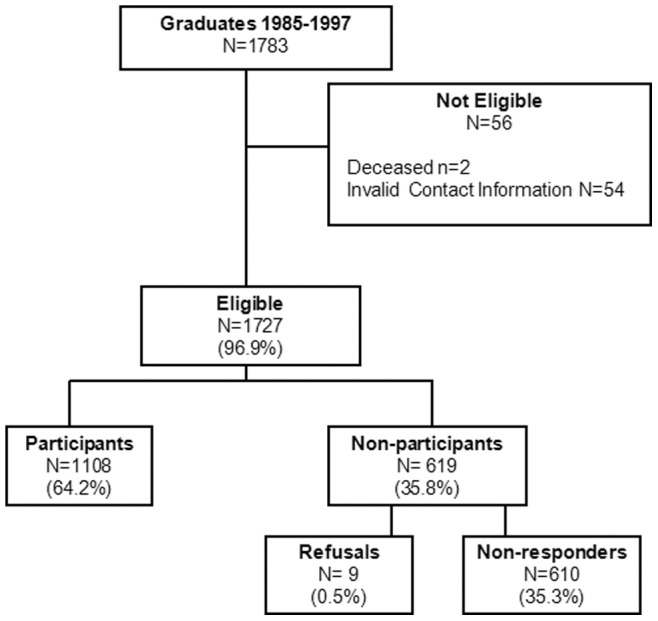
Flow diagram of study participation.

A total of 1783 physicians graduated from medical school from 1985 through 1997. Only 3%, two who were deceased and 54 for whom we had invalid contact information, were excluded from the study sample (Figure 1). A total of 1108 graduates completed the survey yielding a response rate of 64.2%. Physicians who did not participate, compared to those who did, were less likely to be white (70.1% versus 78.7%, p = 0.0002). There were no differences in participation rate by age, gender, undergraduate science major or grade point average, MCAT scores, year of medical school graduation, or completion of a MPH program. Approximately one third of participants provided a current resume, and this data source was not analyzed.

Of the 1108 participants, mean age at the time of medical school graduation was 26.8±2.4 years (44.4±4.4 years at the time of survey completion), 33.9% were women, 81.1% were white, and 17.1% completed a MPH (n = 174 at the time of medical school graduation and n = 16 after medical school graduation). For the 16 graduates completing the MPH after medical school (8% of the physicians with MD/MPH), the mean time to completion of the MPH was 7.1 years (median 5.5 years). Almost half (48.1%) obtained their undergraduate degree from a university in the south, 87.3% had an undergraduate science major, their average undergraduate grade point average was 3.42±0.3 (54.5% with a grade point average ≥3.4), their average MCAT score was 27.6±4.5 (30.1% with a MCAT score ≥30), and the mean time since graduation was 16.4±3.8 years (68.3% ≥15 years since graduation). Medical school graduates with a MPH were more likely to have received their undergraduate degrees from institutions other than the south (Table 1). In addition, those completing MD and MPH degrees were more likely to have fewer than 15 years since graduation from medical school, a finding that tracks the increasing enrollment in the MD-MPH combined program during the later graduation years in the study cohort.

**Table 1:** Baseline Characteristics According to MPH Education Completion among Medical School Graduates.

	MPH* (n = 190)	No MPH** (n = 918)	P value
**Age at school of medicine graduation, n(%)**			
≤26 years	111 (58.4)	559 (60.9)	0.5
>26	79 (41.6)	359 (39.1)	
**Women, n(%)**	75 (39.5)	301 (32.8)	0.08
**White race, n(%)**	156 (82.1)	743 (80.9)	0.71
**Undergraduate university region, n(%)**			
Southern region	61 (32.6)	467 (51.3)	<0.001
Other region ‡	126 (67.4)	444 (48.7)	
**Undergraduate science major, n(%)**	160 (84.2)	807 (87.9)	0.16
**Undergraduate grade point average, n(%)**			
<3.4	85 (44.7)	419 (45.6)	0.82
> = 3.4	105 (55.3)	499 (54.4)	
**MCAT score, n(%)**			
<30	126 (66.3)	649 (70.7)	0.23
> = 30	64 (33.7)	269 (29.3)	
**Time since medical school graduation, n(%)**			
<15 years	74 (38.9)	277 (30.2)	0.02
> = 15 years	116 (61.1)	641 (69.8)	

* Including all public health degree completion: MPH, MSPH or MPH&TM; **Including no public health degree completion.

‡ Other region includes north, midwest, and west coast regions, and outside the US.

MCAT-Medical College Admissions Test; grade point average on 1–4 scale.

Early career choices and outcomes included specialty training, Alpha Omega Alpha induction and board certification. Overall, 18.5% of the respondents completed generalist primary care residency training only. Compared to their MD counterparts who did not receive a MPH, those who completed a MPH had a higher rate of generalist primary care residency training only (31.7% versus 16.4%; *P*<0.001). Specific choices for generalist primary care specialty training by physicians with versus without a MPH were as follows: family medicine (42.4% versus 18.4%, respectively), internal medicine (35.6% versus 46.2%, respectively), and pediatrics (22.0% versus 35.4%, respectively). After multivariable adjustment, physicians with MPH degrees were 1.79 times (95% CI 1.35–2.29) more likely to complete generalist primary care residency training when compared to the graduates with no MPH (Table 2). There was no difference between the two groups with respect to rate of Alpha Omega Alpha induction or board certification.

**Table 2:** Unadjusted and Multivariate-Adjusted Analyses of Early Career Activities Associated with MPH Education Completion among Medical School Graduates.

	Unadjusted			Multivariate	
Early Career Achievements	MPH*(N = 190)	No MPH**(N = 918)	P value	Relative Ris∧ (95% CI)	P value
**Alpha Omega Alpha Member, n (%)**	28 (14.7)	175 (19.1)	0.16	0.73 (0.49, 1.05)	0.09
**Residency training, n (%)**					
Generalist primary care	59 (31.7)	147 (16.4)	<0.001	1.79 (1.35, 2.29)	<.0001
Specialty care	127 (68.3)	749 (83.6)			
**Board certified, n (%)**	184 (96.8)	869 (94.7)	0.21	1.02 (0.98, 1.04)	0.19

* Including all public health degree completion: MPH, MSPH or MPH&TM; **Including no public health degree completion.

∧Estimated from odds ratios and adjusted for age, gender, race, undergraduate university region and time since graduation; CI-confidence interval.

Generalist primary care residency training includes general internal medicine, general pediatrics, and family medicine Specialty care residency training includes all surgical specialties including obstetrics and gynecology, internal medicine and pediatric subspecialties, anesthesiology, radiology, psychiatry, ophthalmology, dermatology, emergency medicine, pathology, neurology, and other.

Significant differences in long-term outcomes were identified among graduates with versus without a MPH (Tables 3 and 4). Medical school graduates with a MPH were 1.81 times (95% CI 1.33–2 37) more likely to be employed in an academic institution, 3.26 times (95% CI 1.89–5.38) more likely to be employed by a government agency, 39.84 times (95% CI 12.13–107.38) more likely to practice public health, 1.59 times (95% CI 1.18–2.05) more likely to practice primary care, and 1.35 times (95% CI 1.02–1.70) more likely to practice in an inner city setting. Medical school graduates with a MPH were less likely to spend more than 75% time in direct patient care 10–20 years after medical school graduation compared to their counterparts without a MPH. Net annual taxable income and satisfaction with their career path were similar between the groups.

**Table 3:** Analyses of Long-term Employment and Medical Practice associated with MPH Education Completion among Medical School Graduates.

	Unadjusted			Multivariate	
Long-term Professional Achievements	MPH* (n = 190)	No MPH** (n = 918)	P value	Relative Ris∧ (95% CI)	P value
**Type of Organization, n(%)**					
Private practice	86 (47.8)	627 (70.5)	<0.001	1.00 (Ref.)	
Academic institution	46 (25.6)	151 (17.0)		1.81 (1.33, 2.37)	0.0003
Governmental agency	24 (13.3)	38 (4.3)		3.26 (1.89, 5.38)	<.0001
Other organization	24 (13.3)	73 (8.2)		1.88 (1.16, 2.93)	0.0111
**Professional Position, n(%)**					
Fee for service clinical practice	70 (39.1)	525 (59.6)	<0.001	1.00 (Ref.)	
Faculty or research appointment	39 (21.8)	125 (14.2)		1.89 (1.32, 2.58)	0.0007
Salaried clinical practice	38 (21.2)	125 (14.2)		1.64 (1.13, 2.29)	0.0106
Administrative	12 (6.7)	63 (7.2)		1.27 (0.66, 2.36)	0.4633
Public health professional	11 (6.1)	2 (0.2)		24.39 (5.15, 99.11)	<.0001
Other	9 (5.0)	41 (4.7)		1.59 (0.73, 3.29)	0.2369
**Type of Medical Practice, n(%)∧∧ #**					
Specialty care	97 (51.6)	674 (74.4)	<0.001	1.00 (Ref.)	
Primary care	52 (27.7)	181 (20.0)		1.59 (1.18, 2.05)	0.0026
Population/Public health	22 (11.7)	3 (0.3)		39.84 (12.13, 107.38)	<.0001
Other	17 (9.0)	48 (5.3)		2.98 (1.70, 4.95)	0.0002
**Practice Setting, n(%)#**					
Suburban	63 (35.6)	436 (49.8)	<0.001	1.00 (Ref.)	
Rural	25 (14.1)	114 (13.0)		1.47 (0.93, 2.21)	0.0974
Inner city	65 (36.7)	265 (30.3)		1.35 (1.02, 1.70)	0.0347
Military	11 (6.2)	27 (3.1)		2.29 (1.08, 4.65)	0.0314
International	8 (4.5)	3 (0.3)		14.62 (3.64, 52.68)	0.0002
Other	5 (2.8)	30 (3.4)		0.75 (0.25, 2.14)	0.5932

* Including all public health degree completion: MPH, MSPH or MPH&TM; **Including no public health degree completion.

∧Estimated from odds ratios and adjusted for age, race, gender, undergraduate university region, years since medical school graduation, and residency training; ∧ not adjusted for residency training.

#additionally adjusted for employed by academic institution and faculty or research appointment; CI-confidence interval.

**Table 4:** Analyses of Long-term Practice Region, % Time in Professional Activities, and Professional Satisfaction associated with MPH Education Completion among Medical School Graduates.

	Unadjusted			Multivariate	
Long-term Professional Achievements	MPH* (n = 190)	No MPH** (n = 918)	P value	Relative Ris∧ (95% CI)	P value
**US Practice Region, n(%)**					
Southern region	87 (46.5)	469 (51.3)	0.0112	1.00 (Ref.)	
Northeast region	14 (7.5)	107 (11.7)		0.51 (0.28, 0.93)	0.0264
Midwest region	12 (6.4)	81 (8.9)		0.56 (0.28, 1.08)	0.0862
West coast region	74 (39.6)	257 (28.1)		1.07 (0.80, 1.39)	0.6196
**Care of Underserved Patients, n(%)**					
No	54 (28.4)	327 (35.6)	0.0572	1.00 (Ref.)	
Yes	136 (71.6)	591 (64.4)		1.07 (0.94, 1.18)	0.2886
**% Time in Patient care, n(%)**					
0∼75%	85 (44.7)	259 (28.2)	<0.001	1.00 (Ref.)	
76∼90%	50 (26.3)	327 (35.6)		0.61 (0.43, 0.83)	0.0014
>90%	55 (28.9)	332 (36.2)		0.62 (0.43, 0.85)	0.0025
**% Time in Administration, n(%)**					
0%	72 (37.9)	292 (31.8)	0.0004	1.00 (Ref.)	
1% ∼ 10%	62 (32.6)	439 (47.8)		0.77 (0.59, 0.97)	0.0244
>10%	56 (29.5)	187 (20.4)		1.15 (0.82, 1.57)	0.4116
**% Time in Teaching, n(%)**					
0%	105 (55.3)	548 (59.7)	0.0144	1.00 (Ref.)	
1% ∼ 10%	55 (28.9)	289 (31.5)		0.89 (0.65, 1.17)	0.4123
>10%	30 (15.8)	81 (8.8)		1.33 (0.74, 2.29)	0.3272
**Very Satisfied with Career Path, n(%)**					
No	70 (38.7)	292 (33.9)	0.2176	1.00 (Ref.)	
Yes	111 (61.3)	570 (66.1)		0.90 (0.77, 1.02)	0.0953
**Net Annual Taxable Income, n(%)**					
<$150k	62 (32.6)	244 (26.6)	0.0098	1.00 (Ref.)	
$150k ∼ $250k	56 (29.5)	215 (23.4)		1.10 (0.79, 1.49)	0.5579
$250k and above	72 (37.9)	459 (50.0)		0.98 (0.77, 1.20)	0.8833

* Including all public health degree completion: MPH, MSPH or MPH&TM; **Including no public health degree completion.

∧Estimated from odds ratios and adjusted for age, race, gender, undergraduate university region, years since medical school graduation, and residency training; CI-confidence interval.

**Table 5:** Analyses of Long-term Research Achievements Associated with MPH Education Completion among Medical School Graduates.

	Unadjusted			Adjusted	
Long-term Research Achievements	MPH* (n = 190)	No MPH**(n = 918)	P value	Relative Ris∧ (95% CI)	P value
**% Time in Research, n(%)**					
<5%	140 (73.7)	780 (85.0)	0.0002	1.00 (Ref.)	0.0005
> = 5%	50 (26.3)	138 (15.0)		2.03 (1.39, 2.80)	
**Type of Research, n(%)**					
No research	87 (45.8)	511 (55.7)	<0.001	1.00 (Ref.)	
Basic	5 (2.6)	40 (4.4)		0.83 (0.29, 2.29)	0.7248
Clinical/Translational	49 (25.8)	334 (36.4)		1.06 (0.80, 1.35)	0.6510
Population/Public health	45 (23.7)	24 (2.6)		8.79 (5.20, 13.82)	<.0001
Other research	4 (2.1)	9 (1.0)		3.18 (0.87, 10.96)	0.0799
**Research Funding Sources, n(%)**					
Not funded	113 (59.5)	648 (70.6)	<0.001	1.00 (Ref.)	
National Institutes of Health/other federal source	21 (11.1)	34 (3.7)		3.11 (1.74, 5.33)	0.0002
Other (includes industry)	56 (29.5)	236 (25.7)		1.35 (1.02, 1.73)	0.0364
**Research Funding as Lead Investigator, n(%)**					
None	136 (71.6)	740 (80.6)	0.0100	1.00 (Ref.)	
$50k – $500k	24 (12.6)	94 (10.2)		1.52 (0.94, 2.36)	0.0838
$500k and above	30 (15.8)	84 (9.2)		2.25 (1.42, 3.38)	0.0007
**Scientific presentations, n(%)**					
0	92 (48.4)	529 (57.6)	0.0002	1.00 (Ref.)	
1∼4	40 (21.1)	230 (25.1)		1.20 (0.85, 1.61)	0.2861
5 and above	58 (30.5)	159 (17.3)		2.31 (1.70, 2.98)	<.0001
**Peer Reviewed Publications, n(%)**					
0	83 (43.7)	475 (51.7)	0.0005	1.00 (Ref.)	
1∼3	40 (21.1)	242 (26.4)		1.21 (0.87, 1.61)	0.2419
4 and above	67 (35.3)	201 (21.9)		2.07 (1.56, 2.60)	<.0001
**Other Publications, n(%)**					
0	115 (60.5)	668 (72.8)	0.0034	1.00 (Ref.)	
1∼3	47 (24.7)	158 (17.2)		1.72 (1.25, 2.28)	0.0013
4 and above	28 (14.7)	92 (10.0)		2.07 (1.27, 3.20)	0.0041

* Including all public health degree completion: MPH, MSPH or MPH&TM; **Including no public health degree completion.

∧Estimated from odds ratios and adjusted for age, race, gender, undergraduate university region, years since medical school graduation, residency training, employment by academic institution, and faculty or research appointment; CI-confidence interval.

With respect to long-term research activities, physicians with MPH degrees were 2.03 times (95% CI 1.39–2.80) more likely to spend >5% time in research, 8.79 times (95% CI 5.20–13.82) more likely to be involved in research in a public health field, 3.11 times (95% CI 1.74–5.33) more likely to have National Institutes of Health or other federal funding, and 2.25 times (95% CI 1.42–3.38) more likely to have ≥ $500,000 in research funding as a lead investigator. Physicians with a MPH were more than twice as likely to have 4 or more peer-reviewed and other publications and to have 5 or more scientific presentations (Table 5).

## Discussion

This study supports the hypothesis that formal public health education via a MPH is associated with specialty training, career choice, and long-term professional outcomes (e.g., professional position, type of medical practice, and research) among medical school graduates. Specifically, physicians with a MPH were more likely to train in generalist primary care specialties (i.e. family medicine) and subsequently to practice primary care and public health, pursue employment in academic institutions or government agencies, engage in population and public health research as a lead investigator, and disseminate more scholarly works than their counterparts without a MPH. Although potential benefits of augmenting medical education with public health training have been previously described [3], [4], [7], [9], [13], this study adds to the literature in that it includes a large sample size, a comparison group of physicians without a MPH, and long-term follow-up to assess professional activities and research implications.

An established pathway to increase the linkages between medical education and public health training is through undergraduate medical education via combined MD/MPH degree programs [21]–[23]. Prior studies have reported a high percentage of MD/MPH graduates selecting a primary care residency; however, there were no comparison groups and the choice of specific primary care specialty varied in different institutions [24], [25]. In our study, physicians with a MPH reported frequent completion of residency training in family practice, a pattern consistent with the US trend for increased residency match in family practice during a similar time period [26]. In addition, a high percentage of medical students in a US school who completed a MPH chose first positions after residency training in academic, governmental or corporate practice settings with more time devoted to non-clinical activities; however, the sample was small (n = 17) and information regarding a comparison group and the long-term practice settings for MD/MPH graduates was lacking [14].

The current study findings indicate that physicians completing a MPH are more engaged in long-term professional activities that strengthen the healthcare workforce through two key mechanisms: 1) primary care and public health practice and 2) research. The first mechanism is important in light of the fact that the US is currently experiencing a shortage of primary care, and public health physicians [4], [26], [27]. Recent reports have revealed a decline in US medical student interest in primary care and a decline in the percentage of students matching in primary care residencies [27]. Although there is limited evidence regarding factors predicting career choice among US medical graduates, what is known suggests that students who express a desire to serve underserved populations, demonstrate altruism and are committed to social responsibility are more likely to go into primary care [28]. It may be that students with these characteristics are more likely to pursue a MPH to acquire the skill set to better serve their community [16]. The availability of programs that foster MD and MPH training may play a role in enhancing the skill set for primary care and public health practice. Although some earlier reports noted that a high percentage of graduates of MD/MPH programs pursue careers in primary care and public health-related fields [14], [24], [25], this analysis reveals a higher rate of active practice in primary care and public health 10–20 years after graduation by physicians with a MPH than by their counterparts with no MPH: 27% and 11.9% of the medical school graduates with a MPH were engaged in primary care and public health practice, respectively, versus 20.1% and 0.3%, respectively, of the graduates with no MPH. Of note, the association of MPH education was stronger for public health practice (RR  = 39.84) than for primary care practice (RR  = 1.59). The second mechanism, research, is a somewhat unexpected but not surprising finding. Unlike physicians completing MD-PhD degree programs [29], [30] the goal for most physicians completing a MPH was not to develop as physician scientists. However, the content of public health courses such as biostatistics, epidemiology and program evaluation provide a research foundation, and the exposure to faculty engaged in public health research provide role models [31]–[33] for establishing conduct of research as a career goal. It is noteworthy that the association between MPH education and research achievements was strong even though a higher percentage of physicians completing a MPH versus no MPH had less than 15 years since graduation (38.9% versus 30.2%, respectively) providing less time for professional achievements. In addition, a higher percentage of physicians with a MPH pursued a faculty or research appointment (reflecting a choice for an academic career) compared to their counterparts without a MPH (21.8% versus 14.2%, respectively). This finding is consistent with results of a published systematic review revealing an association between completion of a MD with a graduate degree (i.e, masters or PhD) and a career in academic medicine [32]. Of note, professional activities for physicians in academic settings are typically balanced with patient care, teaching and research. MD/MPH graduates were more likely than the MD/no MPH to spend ≥5% time in research (26.3% versus 15.0%, respectively). A subsequent analyses of physicians spending ≥5% versus <5% time in research revealed higher prevalence of scientific presentations, peer-reviewed publications, and other publications (data not shown). After multivariable adjustment, physicians with a MPH versus no MPH were more likely to receive grant funding from the National Institutes of Health or other federal source, publish more peer reviewed and other publications and present more scientific papers. The engagement in and dissemination of research findings is important for informing the healthcare community about generalizable knowledge to improve health.

These study findings can be considered in a broader context. Although the traditional structure of undergraduate medical education and postgraduate medical training and work in developed countries such as the UK differs from that of the US system, the gaps in public health teaching to medical students and the need for a strengthened public health workforce are similar [8], [9], [34]. The international literature suggests a frequent concern securing and maintaining medical students’ interest in public health [9], [35]–[38]. Like the decline in interest in primary care in the US, there has been a substantial shift away from general practice as a career choice in the UK [39]–[41]. Factors influencing career choice have been identified and include experiences of the chosen subject in medical school, a particular teacher or department or inclination before medical school [31]. Comparisons with other specialties in the UK showed that doctors in public health chose their specialty relatively late after qualification [42]. Timing of exposure to public health training may influence career choice, and opportunities to combine undergraduate medical education with formal public health training may exist. A study evaluating career choice of medical students who completed a research-based honors year in public health and epidemiology revealed 19% (37/195) of these medical school graduates chose an academic career [43]. Despite variances in educational structure for physicians in different countries, the current study supports exploration of formal public health training via completion of a MPH degree on the impact of career choice and professional practice in international medical education systems.

The study results should be interpreted in light of its limitations. Because the study was based on a retrospective cohort design, causal inferences cannot be made. The participants were from a single institution in the US with a longstanding commitment to medical and public health education and may not represent all physicians exposed to MPH education. As in any survey study, a potential weakness is non-responder bias. It is possible that non-responders were different from responders with respect to the association between MPH completion and career choice, specialty training, and long term outcomes. Non-participants were more likely to be nonwhite than participants; thus, the results may under represent nonwhites. The self-report survey is subject to recall and social desirability biases. However, any such biases were likely to have been similar for both groups. Lastly, the cohort included medical school graduates between 1985 and 1997, a time period prior to the decline in primary care specialty choice beginning in 1998 [26]. However, 42.9% (78/182) of the medical school graduates with MPH degrees from this same institution over the last 5 years (2007–2011) has matched in generalist primary care residency programs.

The current analysis has several notable strengths. The data were obtained from a large population of medical school graduates and allowed comparison between physicians with versus without a MPH degree while minimizing potential confounding due to program and regional differences. To assess the impact of formal public health training, all physicians with a MPH were grouped together irrespective of the timing of the MPH degree relative to the MD degree. (In this study, only 8% of the MD/MPH graduates completed the MPH after medical school graduation. For these graduates, it may be that their clinical work led the graduates to want to acquire skills, via a MPH, to better manage their working environment. Of note, the results in this study were similar when the analysis was restricted to participants who completed the MPH concurrently with the MD versus the MD/no MPH-data not shown). In addition, results were similar when comparing MD-MPH to MD-no MPH or other advance degree (data not shown). The characteristics of our cohort are similar to that of all US medical school students [44] and reveal diversity with respect to demographics, region of undergraduate education, and region of practice. The 64.2% response rate was relatively high for a practicing physician cohort. The extensive nature of our data collection including objective outcomes, use of a standardized survey protocol, adherence to quality control procedures, and inclusion of a comparison group permitted conduct of a more comprehensive analysis of the association between completion of a MPH and long-term professional achievements among physicians than previously possible.

### Conclusion

Formal public health education via a MPH degree among US-trained physicians was strongly associated with early career choices and long-term professional achievements in public health and primary care practice and research. These findings support the recommendations of the Institute of Medicine to increase the proportion of physicians with formal public health training and could inform a research agenda to more fully explore the extent to which MD and MPH education goals are being met in the US allopathic medical education system. Augmenting medical education with public health training through undergraduate medical education (e.g, via combined MD/MPH degree programs), post graduate education (e.g., residency training in preventive medicine), and mid-career public health education provides an infrastructure to build a strong physician workforce by facilitating collaborations between schools of public health and medicine and by producing graduates who are sensitive to the interplay between health promotion, disease prevention, and clinical care and committed to addressing knowledge gaps in these areas through research.
